# Association of fermented dairy consumption with asthma and wheeze risk in children: a systematic review and meta-analysis of observational studies

**DOI:** 10.3389/fnut.2026.1860222

**Published:** 2026-07-21

**Authors:** Bing Tian, Lijie Hou, Ling Zhang, Zhuang Wang, Xiaofei Xie, Dongze Li, Yongji Wang

**Affiliations:** 1 College of Traditional Chinese Medicine, Changchun University of Chinese Medicine, Changchun, Jilin, China; 2 Department of Pediatric Internal Medicine One, Affiliated Hospital of Changchun University of Chinese Medicine, Changchun, Jilin, China; 3 Department of Cardiovascular Medicine, China-Japan Union Hospital of Jilin University, Changchun, Jilin, China

**Keywords:** asthma, children, fermented dairy, meta-analysis, systematic review, wheeze

## Abstract

**Objective:**

This study aimed to systematically assess the association between fermented dairy consumption and the risk of asthma and wheeze in children.

**Methods:**

Following a protocol registered with PROSPERO (CRD420251269745), a systematic search was conducted in PubMed, Embase, Web of Science, and the Cochrane Library up to January 2026 to identify observational studies reporting relevant effect estimates. A random-effects model was used to pool odds ratios (ORs) with 95% confidence intervals (CIs). Subgroup analyses were performed based on study design, outcome phenotype, product type, and timing of exposure. Sensitivity analyses, including Hartung–Knapp adjustment, were conducted to assess robustness.

**Results:**

A total of 11 studies (119,372 participants) were included. In the primary DerSimonian–Laird random-effects analysis, fermented dairy consumption was associated with a reduced risk of the composite outcome of asthma/wheeze in children (OR = 0.72, 95% CI: 0.55–0.95), with substantial heterogeneity (*I*^2^ = 77.0%). However, this association was not retained after Hartung–Knapp adjustment (OR = 0.72, 95% CI: 0.52–1.01; *p* = 0.054). Subgroup analyses indicated that this association was largely influenced by cross-sectional studies (OR = 0.51, 95% CI: 0.33–0.78), whereas no statistically significant association was found in cohort studies (OR = 0.91, 95% CI: 0.72–1.16). Additional subgroup analyses showed stratum-specific differences, but tests for subgroup differences were not statistically significant. Egger’s test did not indicate statistically significant publication bias. The overall certainty of evidence was very low.

**Conclusion:**

The current evidence does not support a robust association between fermented dairy consumption and the risk of asthma or wheeze in children. The observed potential association may be influenced by methodological factors, particularly study design. Well-designed, large-scale prospective studies are warranted to further clarify this association.

**Systematic review registration:**

https://www.crd.york.ac.uk/PROSPERO/view/CRD420251269745, identifier: CRD420251269745.

## 1 Introduction

Asthma is a major noncommunicable disease worldwide posing a significant public health burden (1). Wheeze, particularly during infancy and the preschool years, is a common respiratory symptom that often recurs in individual children and is associated with a significantly increased risk of subsequent asthma development (2, 3). Despite substantial progress in pharmacological treatment over the past decades, the global prevalence of childhood asthma and wheezing disorders remains high (4). Consequently, increasing attention has been directed toward modifiable early-life dietary factors that may influence the risk of childhood asthma and wheeze by modulating immune development and airway inflammation (5–7).

Fermented dairy products, such as yogurt and cheese, are widely consumed worldwide and are considered important dietary sources of various beneficial components, including probiotics, calcium, vitamins, and bioactive peptides, which may enhance the nutritional value of dairy products and confer multiple health benefits (8–11). During fermentation, microbial activity can transform the dairy matrix and generate bioactive or more bioavailable end-products, including milk-derived peptides, organic acids, and other microbial metabolites (12, 13). These fermentation-related compounds may plausibly influence host immune regulation through effects on epithelial barrier function, systemic inflammatory signaling, and gut microbiota–derived short-chain fatty acid signaling pathways, which have been linked experimentally to allergic airway inflammation (14, 15). Experimental and epidemiological evidence suggests that modulation of the gut microbiota by diet may influence immune regulation through the “gut–lung axis” (16–18). Through this pathway, diet-related microbial and metabolic interactions may contribute to the development or prevention of allergic and respiratory diseases (19, 20). Given these biological mechanisms, a growing number of epidemiological studies have investigated the association between fermented dairy consumption and childhood asthma and wheeze. However, findings regarding its relationship with asthma-related outcomes in children remain inconsistent, and no consensus has been reached.

Although several observational studies have investigated the association between fermented dairy consumption and the risk of childhood asthma and wheeze, findings vary across study designs (e.g., cohort studies versus cross-sectional studies) (21, 22). Moreover, differences in the timing of exposure (e.g., maternal intake during pregnancy versus childhood intake) may contribute to inconsistent findings (23, 24). Furthermore, the types of fermented dairy products examined in previous studies differ, including yogurt, fermented milk, and cheese, yet the potential differential effects among these product types have not been systematically compared (25–27). Notably, existing studies also lack consistency in distinguishing wheeze from physician-diagnosed asthma as distinct respiratory phenotypes (28–31). These limitations hinder a clear interpretation of the current evidence, highlighting the need for a more comprehensive synthesis of the available data.

This study conducted a systematic review and meta-analysis of observational studies to evaluate the association between fermented dairy consumption and the risk of childhood asthma and wheeze. Furthermore, we performed stratified analyses according to factors such as respiratory phenotype, product type, timing of exposure, and study design, aiming to more comprehensively clarify the potential relationships reflected by the current evidence and to inform future research and clinical practice.

## 2 Methods

### 2.1 Study design and registration

This systematic review and meta-analysis was conducted in accordance with the Preferred Reporting Items for Systematic Reviews and Meta-Analyses (PRISMA 2020) statement (32). The study protocol was registered with the International Prospective Register of Systematic Reviews (PROSPERO) under registration number CRD420251269745.

### 2.2 Search strategy

A systematic search was performed in PubMed, Embase, Web of Science, and the Cochrane Library from database inception to January 2026. The search strategy included terms related to fermented dairy products (e.g., “fermented dairy,” “yogurt,” “fermented milk,” “cheese”) with respiratory outcome terms (“asthma,” “wheeze”), using both Medical Subject Headings (MeSH) and free-text terms. Only studies published in English were included. Detailed search strategies for each database are provided in Supplementary Tables S1–S4.

### 2.3 Inclusion and exclusion criteria

The inclusion and exclusion criteria (Table 1) were defined based on the PICO framework (33). Fermented dairy products were defined as dairy products produced through microbial fermentation, including yogurt, cheese, and fermented milk products. These products were pooled as a broad exposure category because they share the defining feature of microbial fermentation of dairy substrates, and because several included studies reported composite fermented dairy exposures. Asthma and wheeze were pooled as a composite primary outcome because wheeze is frequently used in pediatric epidemiological studies as a symptom-based outcome related to asthma risk, and the limited number of available studies precluded fully separate primary analyses.

**Table 1:** Inclusion and exclusion criteria.

Element	Inclusion and exclusion criteria for studies
Population	Children (aged ≤18 years), regardless of sex, race, or geographic region. Studies assessing maternal consumption of fermented dairy products during pregnancy in relation to the risk of asthma/wheeze in offspring (with offspring aged ≤18 years) were also included.
Intervention/Exposure	Fermented dairy products (including yogurt, cheese, fermented milk, etc.) consumed as part of the usual diet. The timing of exposure could be during pregnancy (maternal intake) or during childhood (the child’s own intake). No restrictions were placed on the frequency, dose, or bacterial strains involved in fermentation.
Comparator	Participants with no or the lowest intake of fermented dairy products (e.g., lowest quartile, non-consumers, etc.). In observational studies, comparisons across different intake levels (e.g., quartiles) were allowed.
Outcomes	Asthma or wheeze, defined using validated or standardized criteria, including physician diagnosis, standardized questionnaires such as the ISAAC, or International Classification of Diseases codes.
Study design	Observational studies (cohort studies, case–control studies, cross-sectional studies) were included. Randomized controlled trials, reviews, conference abstracts, animal studies, *in vitro* studies were excluded. Only studies published in English were included.

### 2.4 Data extraction

Two investigators independently screened the titles, abstracts, and full texts of retrieved records after removing duplicates. Data were extracted independently by two reviewers using a standardized extraction form in accordance with established systematic review guidelines (34). Extracted information included study characteristics (first author, year, country/region), study design, sample size, age range, exposure assessment methods, outcome definitions, and covariates adjusted for in the analyses. Any discrepancies in study selection or data extraction were resolved by discussion with a third reviewer.

As some primary studies reported multiple effect estimates for different exposures (e.g., yogurt and cheese) or exposure windows (e.g., pregnancy and childhood), a pre-specified hierarchy for selecting effect estimates was established prior to data extraction to ensure that each study contributed only one effect estimate to the main analysis and to avoid double counting of participants. The hierarchy was as follows: (1) childhood exposure was prioritized over maternal pregnancy exposure; (2) wheeze outcomes were prioritized over physician-diagnosed asthma due to their closer reflection of early respiratory manifestations in children; (3) when neither was available, the primary reported exposure and outcome were used. For subgroup analyses, all available effect estimates were extracted according to predefined strata (exposure window, product type, and outcome definition) to explore heterogeneity. In these analyses, multiple effect estimates from a single study were allowed, and a random-effects model was applied. When multiple estimates from the same study contributed to different subgroup strata, these estimates were treated as stratum-specific estimates rather than fully independent observations. Because within-study correlations among these estimates were not reported and the number of estimates within each subgroup was limited, multilevel or robust variance models were not applied; therefore, subgroup analyses were considered exploratory and interpreted cautiously (35).

### 2.5 Quality assessment

We assessed the quality of cohort and case–control studies using the Newcastle–Ottawa Scale (NOS) (36), and a modified version of the NOS was applied for cross-sectional studies (37). Quality assessment was independently performed by two reviewers, and any discrepancies were resolved through discussion with a third reviewer. Studies scoring 7 points or higher were classified as high quality, those scoring 5 to 6 points as moderate quality, and those scoring 4 points or lower as low quality.

### 2.6 Statistical analysis

The odds ratio (OR) was used as the uniform effect size metric. For relative risks (RRs) and hazard ratios (HRs), these were approximated as ORs for pooling under the rare event assumption (outcome incidence <10%) (38). Cochran’s *Q* test (with a significance threshold of *p* < 0.1) was used to determine whether between-study heterogeneity was statistically significant, and the *I*^2^ statistic was used to quantify the degree of heterogeneity (39), with values of 25, 50, and 75% representing low, moderate, and high heterogeneity, respectively (40). For low heterogeneity (*p* ≥ 0.1 and *I*^2^ ≤ 50%), a fixed-effects model (Mantel–Haenszel method) was used to pool effect estimates; for moderate or high heterogeneity (*p* < 0.1 or *I*^2^ > 50%), a random-effects model (DerSimonian–Laird method) was applied (41). As an additional sensitivity analysis of the random-effects inference, the Hartung–Knapp adjustment was applied using the DerSimonian–Laird estimate of between-study variance. This analysis was conducted for the primary pooled analysis and for the study-design subgroup analyses. Hartung–Knapp confidence intervals and *p* values were calculated using a t distribution with k − 1 degrees of freedom, where k denotes the number of studies included in each analysis. Prespecified subgroup analyses included respiratory phenotype, product type, timing of exposure, and study design. In addition to the prespecified subgroup analyses, an exploratory subgroup analysis by geographic region was performed to further examine potential sources of between-study heterogeneity. Because several individual regions were represented by only one or a few studies, studies were grouped into European and non-European categories to avoid unstable single-study strata. This analysis was considered exploratory and was interpreted cautiously. Sensitivity analyses were conducted by excluding one study at a time to test the robustness of the results, and by restricting the pooled analysis to studies reporting ORs (i.e., excluding those reporting RRs or HRs) to assess the impact of the approximation approach (35, 39). Additional sensitivity analyses were conducted to assess the influence of methodological quality and confounder adjustment. First, the analysis was restricted to studies with a Newcastle–Ottawa Scale score of 7 or above. Second, studies with minimal confounder adjustment, defined as studies reporting univariate estimates only or adjusting for only a very limited set of covariates, were excluded. These analyses were performed using the DerSimonian–Laird random-effects model for comparability with the primary analysis. Publication bias was assessed using funnel plots and Egger’s regression test (42). When funnel plot asymmetry was detected, the trim-and-fill method was used to estimate the number of missing studies (43). The Grading of Recommendations Assessment, Development and Evaluation (GRADE) system was used to grade the certainty of evidence for the primary outcome of “fermented dairy consumption and the risk of childhood asthma/wheeze.” The initial evidence from observational studies was considered low certainty, and was downgraded based on risk of bias, inconsistency, imprecision, publication bias, and indirectness, with the final certainty rated as high, moderate, low, or very low (44). All statistical analyses were performed using Stata software (version 18.0). A two-sided *p* < 0.05 was considered statistically significant for all analyses, except for Cochran’s *Q* test where *p* < 0.1 was used to indicate statistically significant heterogeneity.

## 3 Results

### 3.1 Study selection and characteristics

**Figure 1:**
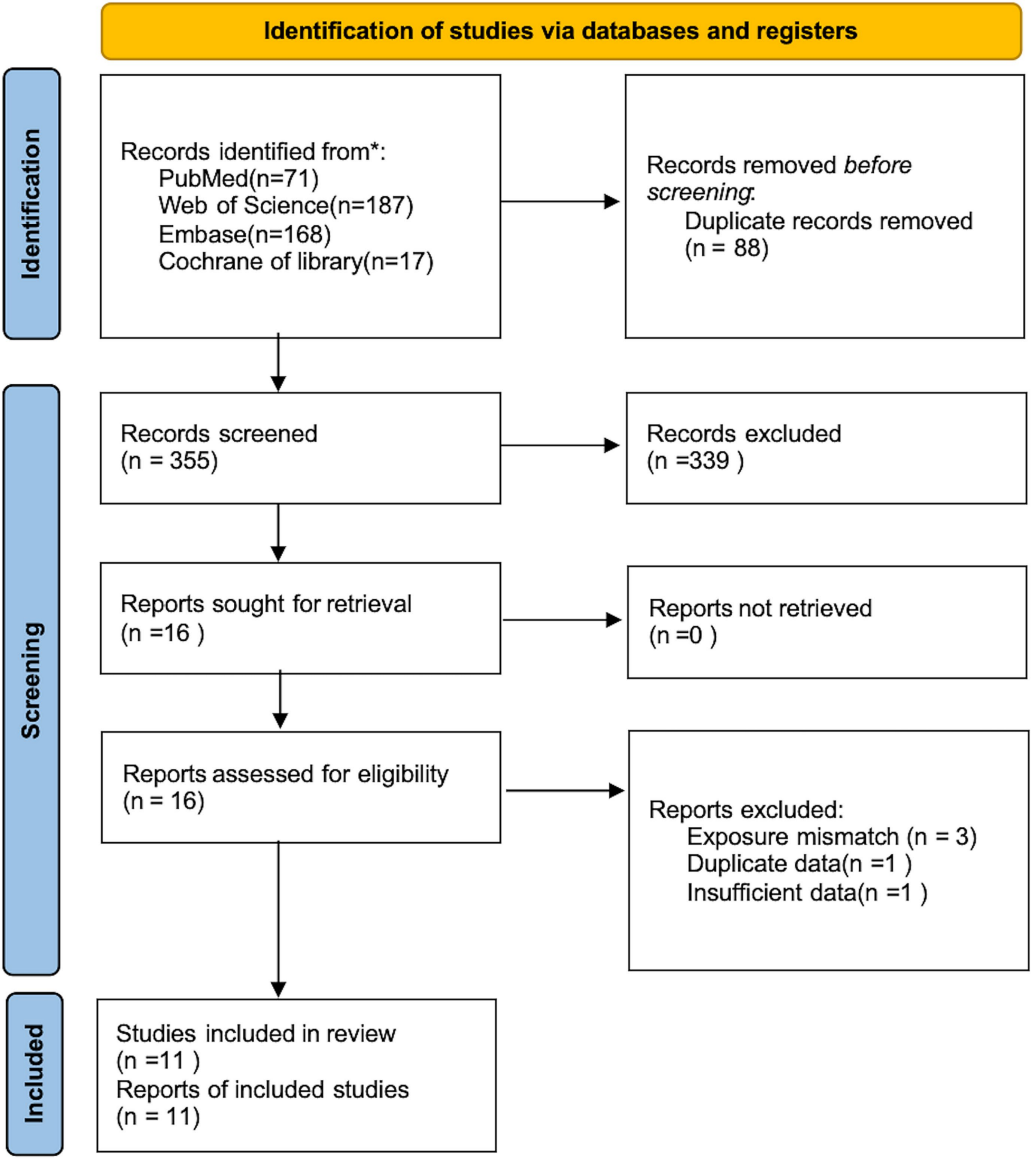
Flow diagram of database searches and results.

A total of 443 records were identified through the database searches. After removal of duplicates and screening of titles, abstracts, and full texts according to the predefined inclusion and exclusion criteria, 11 observational studies were finally included in the final analysis (21–31), comprising a total of 119,372 participants (Figure 1). The included studies originated from multiple regions, including Europe, Asia, Oceania, and Africa. In terms of study design, seven cohort studies and four cross-sectional studies were included. Dietary exposure was primarily assessed using food frequency questionnaires or dietary recall methods. Study outcomes included physician-diagnosed asthma and parent-reported wheeze, which were ascertained using standardized instruments, such as the ISAAC questionnaire, study-specific questionnaires, or national medical registries. Most of the included studies adjusted for key potential confounders, including age, sex, parental smoking, socioeconomic status, and family history of allergy. The main study characteristics are summarized in Table 2.

**Table 2:** Characteristics of the included studies.

Author	Year	Country	Study type	Sample size	age range	Exposure	Exposure assessment	Outcome assessment	Adjusted covariates
Koivusaari	2021	Finland	cohort study	3,053	5 years (age at outcome assessment)	Fermented milk products/ Cheese	3-day food records at multiple time points	ISAAC + IgE	Parental atopy, sex, gestational age, energy intake
K. Wickens	2002	New Zealand	Cross-sectional study	293	7–10 years	Yogurt/Cheese	ISAAC-based parent questionnaire	ISAAC questionnaire	Sex, ethnicity, maternal education, family history of allergy, smoking, antibiotic use
Jamalvandi	2024	Iran	Cross-sectional study	7,667	6–8 years and 13–14 years	Fermented milk products	GAN questionnaire	GAN/ISAAC questionnaire	age, sex,TV/computer use, BMI
Bertelsen	2014	Norway	cohort study	40,614	6–36 months (main outcome at 36 months)	Fermented milk products	FFQ at gestation week 22 (probiotic milk/yogurt)	Parent-reported questionnaires	Maternal age, smoking, education, BMI, energy intake, breastfeeding, maternal atopy, sex
Levin	2019	South Africa	Cross-sectional study	1,583	12–36 months	Fermented milk products	Interviewer-administered questionnaire	Clinical exam + SPT + OFC	None (univariate analysis only)
Miyake	2014	Japan	cohort study	1,354	23–29 months	Yogurt/Cheese	Self-administered DHQ (150 items, previous month)	ISAAC + physician diagnosis	Maternal age, education, parental atopy, smoking, breastfeeding, sex
Miyake	2009	Japan	cohort study	763	16–24 months	Yogurt/Cheese	Self-administered DHQ (150 items, previous month)	ISAAC-based wheeze/eczema	Maternal age, education, parental atopy, smoking, breastfeeding, sex
Fsadni	2018	Malta	Cross-sectional study	191	9–11 years	Yogurt	ISAAC-based parent questionnaire	ISAAC questionnaire	paternal level of education
Stampfli	2022	Austria, Finland, France, Germany, Switzerland.	cohort study	1,014	12–24 months (diet assessment); followed to 6 years	Yogurt/Cheese	Questionnaires at 1.5 and 2 years (food items)	Physician diagnosis + IgE	Sex, parental atopy, farming, breastfeeding, pet exposure
Maslova	2012	Denmark	cohort study	61,909	18 months and 7 years	Yogurt	Validated 360-item semi-quantitative FFQ	Interview + ISAAC + registries	Maternal age, smoking, BMI, breastfeeding, parental atopy, energy intake
Nicklaus	2019	Norway	Cohort study	931	Cheese consumption assessed at 18 months; allergic outcomes followed up to 6 years	Cheese	Monthly diaries + 18-month questionnaire (cheese types/frequency)	Physician diagnosis + IgE	Center, farmer, parental atopy, maternal education, breastfeeding

### 3.2 Quality assessment of included studies

The methodological quality of the included studies, as assessed using the NOS, ranged from 6 to 9, indicating moderate to high quality (Supplementary Table S5, S6). Overall, the quality of the included studies was considered acceptable for meta-analysis.

### 3.3 Primary analysis: overall association between fermented dairy consumption and childhood asthma/wheeze

**Figure 2:**
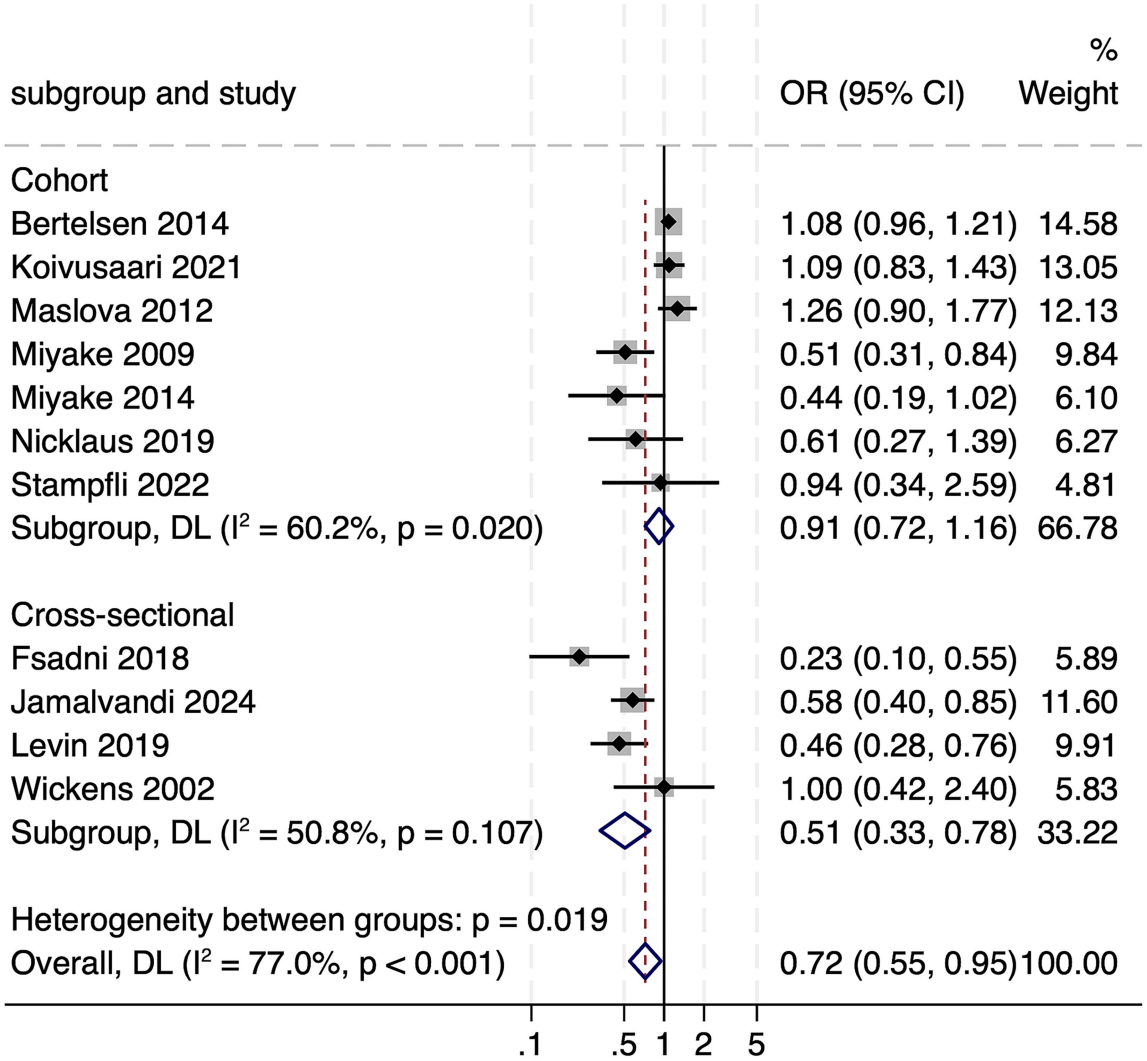
Forest plot of the overall association between fermented dairy consumption and the risk of childhood asthma/wheeze, with subgroup analysis by study design.

The heterogeneity test indicated substantial heterogeneity across studies (*I*^2^ = 77.0%, *p* < 0.001), prompting the use of a random-effects model was applied to account for between-study variability. The pooled analysis showed that fermented dairy consumption was associated with a reduced risk of the combined outcome of asthma and wheeze in children (OR = 0.72, 95% CI: 0.55–0.95). To further explore potential sources of heterogeneity, the following subgroup analyses were performed (Figure 2).

### 3.4 Subgroup analyses

#### 3.4.1 Stratification by study design

Subgroup analysis by study design (Figure 2) showed that among cohort studies (n = 7), no statistically significant association was observed between fermented dairy consumption and childhood asthma/wheeze (OR = 0.91, 95% CI: 0.72–1.16; *I*^2^ = 60.2%). By comparison, cross-sectional studies (*n* = 4) showed a significant association with a reduced risk of childhood asthma/wheeze (OR = 0.51, 95% CI: 0.33–0.78; *I*^2^ = 50.8%). The test for subgroup differences indicated a statistically significant difference between the two study designs (*p* = 0.019), suggesting that study design may be a potential source of heterogeneity.

#### 3.4.2 Stratification by respiratory phenotype

**Figure 3:**
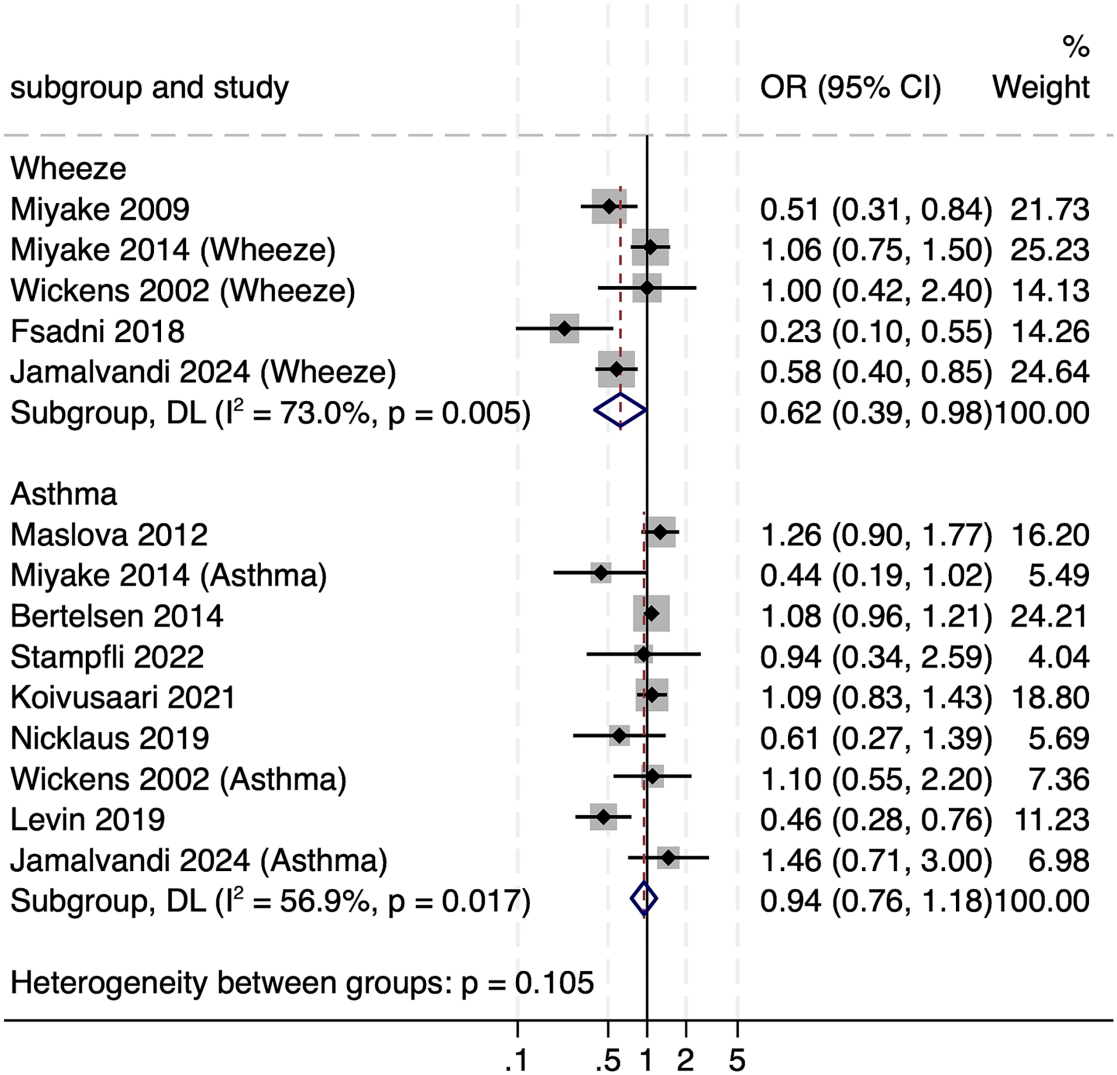
Forest plot of subgroup analysis by respiratory phenotype.

Subgroup analysis by respiratory phenotype (Figure 3) showed that the pooled estimate was stronger for wheeze (OR = 0.62, 95% CI: 0.39–0.98; *I*^2^ = 73.0%) than for physician-diagnosed asthma (OR = 0.94, 95% CI: 0.76–1.18; *I*^2^ = 56.9%). However, the test for subgroup differences was not statistically significant (*p* = 0.105).

#### 3.4.3 Stratification by type of dairy product

**Figure 4:**
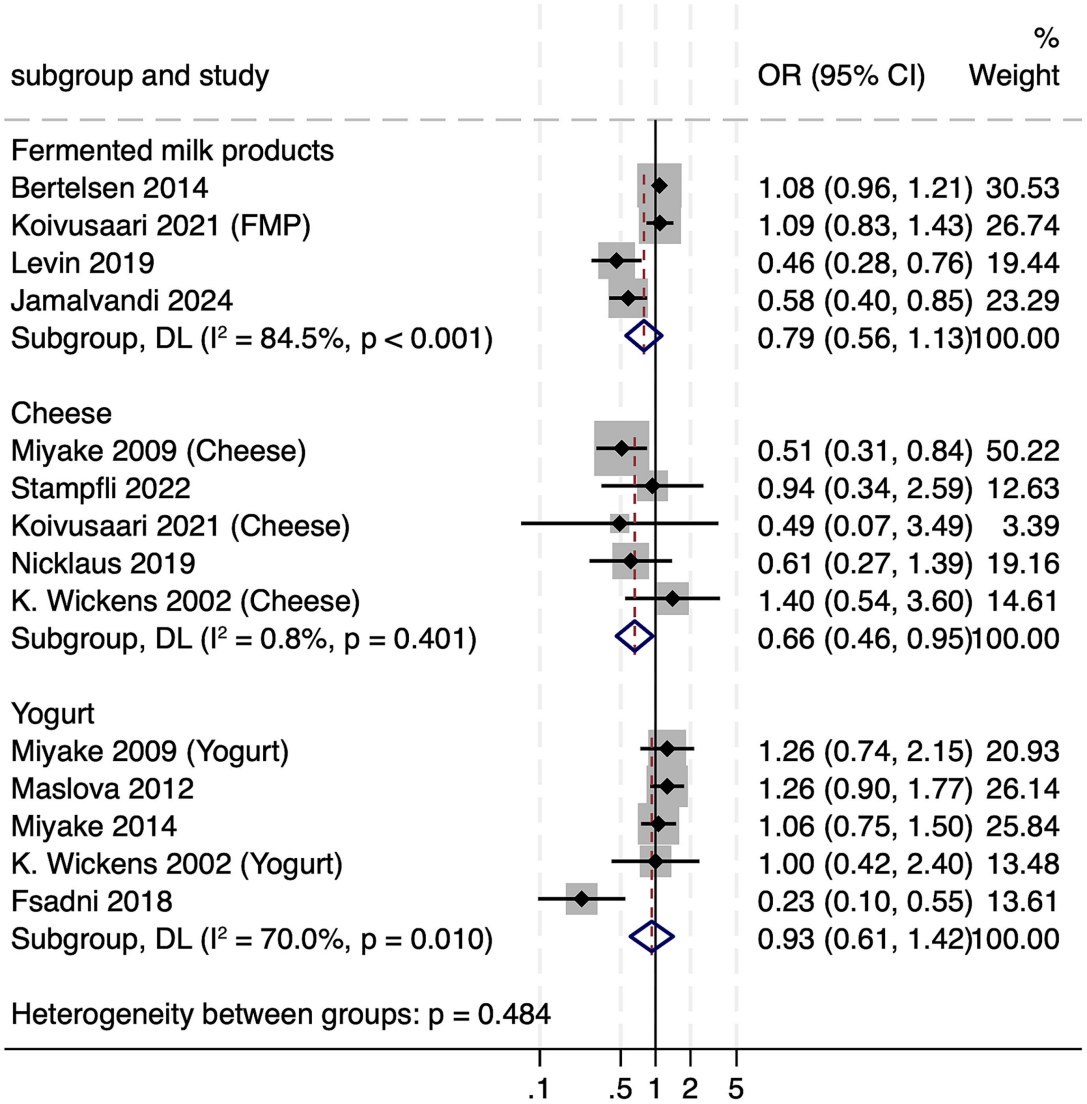
Forest plot of subgroup analysis by type of fermented dairy product.

Subgroup analysis by type of dairy product (Figure 4) showed that cheese consumption was associated with a reduced risk (OR = 0.66, 95% CI: 0.46–0.95; *I*^2^ = 0.8%); yogurt consumption showed no significant association (OR = 0.93, 95% CI: 0.61–1.42; *I*^2^ = 70.0%); nor did fermented milk products (OR = 0.79, 95% CI: 0.56–1.13; *I*^2^ = 84.5%). The test for subgroup differences gave a *p*-value of 0.484, indicating no statistically significant difference in effect estimates across the three product types.

#### 3.4.4 Stratification by timing of exposure

**Figure 5:**
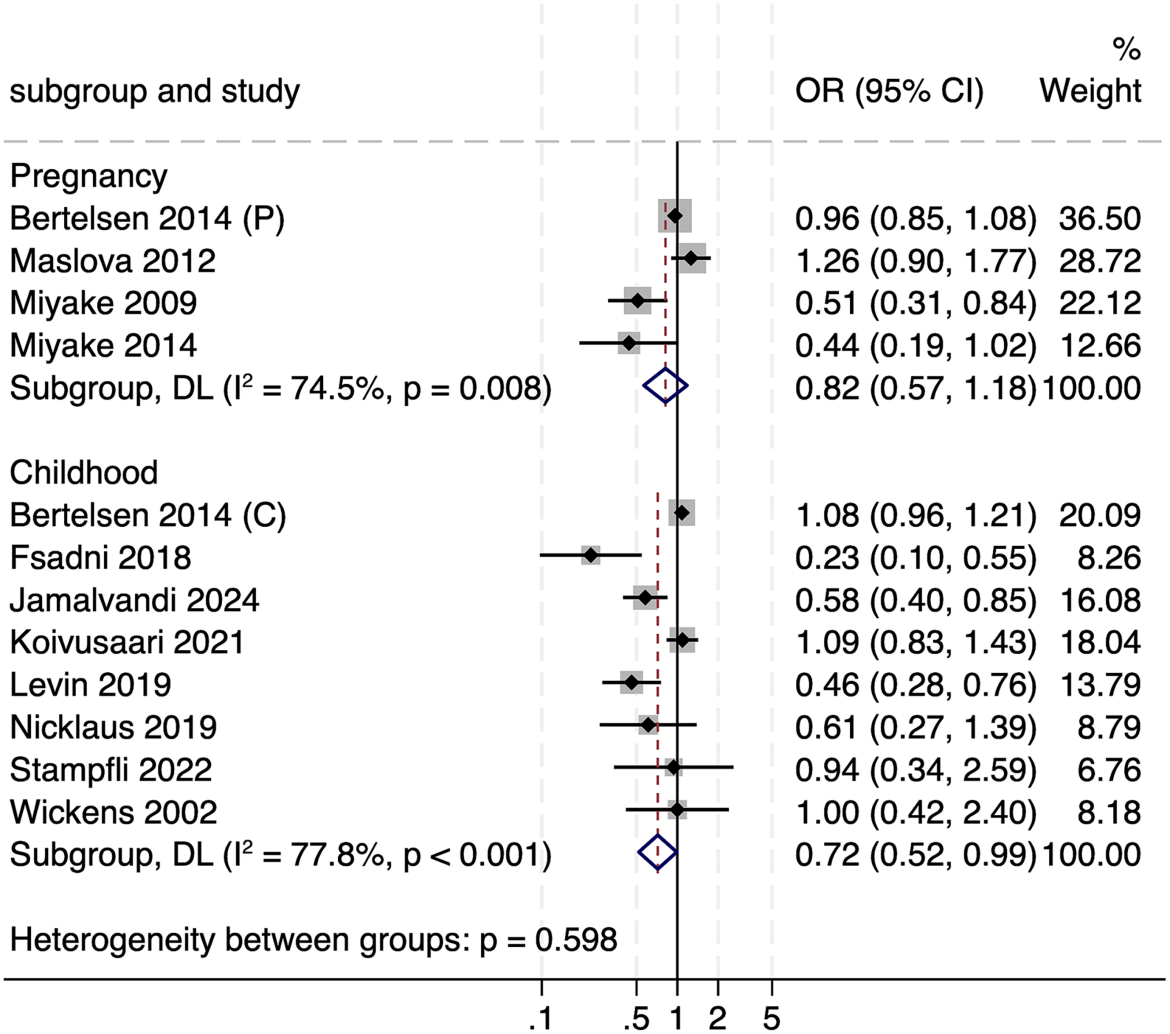
Forest plot of subgroup analysis by timing of exposure.

Subgroup analysis by timing of exposure (Figure 5) showed that childhood exposure to fermented dairy products was associated with a reduced risk (OR = 0.72, 95% CI: 0.52–0.99); while prenatal exposure during pregnancy showed no statistically significant association (OR = 0.82, 95% CI: 0.57–1.18). The test for subgroup differences gave a *p*-value of 0.598, indicating no evidence of a statistically significant difference in effect estimates between the two exposure windows.

#### 3.4.5 Stratification by geographic region

Exploratory subgroup analysis by geographic region (Supplementary Figure S5) showed no statistically significant association among European studies (OR = 0.94, 95% CI: 0.72–1.24; *I*^2^ = 66.3%), whereas non-European studies showed a stronger inverse association (OR = 0.54, 95% CI: 0.43–0.69; *I*^2^ = 0.0%). The test for subgroup differences was statistically significant (*p* = 0.003), suggesting that geographic region may be a potential source of heterogeneity; however, this finding should be interpreted as exploratory.

### 3.5 Sensitivity analyses

#### 3.5.1 Leave-one-out analysis

A leave-one-out sensitivity analysis was performed to assess the robustness of the pooled results. The overall effect estimate (OR) remained relatively stable; however, after excluding Miyake 2009, Miyake 2014, Fsadni 2018, Jamalvandi 2024 or Levin 2019, the 95% confidence interval of the pooled estimate included or closely approached the null value (OR = 1), suggesting that the overall result was less stable and that its statistical significance may be influenced by individual studies (Supplementary Figure S1).

#### 3.5.2 Exclusion of studies reporting RR or HR

To evaluate the impact of approximating RRs and HRs as ORs, two studies that reported RRs or HRs (Koivusaari 2021, Bertelsen 2014) were excluded, and a re-analysis was performed using only studies reporting ORs. The pooled OR was 0.61 (95% CI: 0.43–0.87, *I*^2^ = 68.4%), which remained statistically significant, suggesting that the findings were robust to the effect size conversion approach (Supplementary Figure S2).

#### 3.5.3 Hartung–Knapp sensitivity analysis

A Hartung–Knapp sensitivity analysis was performed to evaluate the robustness of the random-effects inference (Supplementary Table S7). In the primary analysis, the point estimate remained unchanged (OR = 0.72), but the 95% CI widened from 0.55–0.95 to 0.52–1.01 (*p* = 0.054; *q* = 1.18; df = 10), and the association was no longer statistically significant. In the study-design strata, the cohort subgroup remained non-significant (OR = 0.91, 95% CI: 0.64–1.30; *p* = 0.555), whereas the cross-sectional subgroup lost statistical significance after Hartung–Knapp adjustment (OR = 0.51, 95% CI: 0.23–1.13; *p* = 0.073). These findings suggest that the statistical significance of the primary pooled estimate and the cross-sectional subgroup estimate was sensitive to the random-effects inference method.

#### 3.5.4 Sensitivity analysis by study quality and confounder adjustment

Additional sensitivity analyses were performed to assess the influence of study quality and confounder adjustment (Supplementary Table S8). When restricted to studies with a Newcastle–Ottawa Scale score ≥7, the pooled association was attenuated and no longer statistically significant (OR = 0.78, 95% CI: 0.61–1.01; *p* = 0.058; *I*^2^ = 72.6%). After excluding minimally adjusted studies [Levin et al. (25) and Fsadni et al. (28)], the pooled estimate was further attenuated and remained non-significant (OR = 0.85, 95% CI: 0.67–1.07; *p* = 0.172; *I*^2^ = 66.4%) (Supplementary Figure S4). These findings suggest that the overall inverse association was attenuated after excluding studies with lower methodological quality or minimal confounder adjustment, indicating that the primary result was sensitive to study quality and confounder control.

### 3.6 Publication bias

Visual inspection of the trim-and-fill funnel plot showed slight asymmetry, with a slight excess of studies on the left (protective effect) side; however, the trim-and-fill method did not identify any missing studies, and the adjusted pooled effect estimate was consistent with the original result (Supplementary Figure S3). Egger’s regression test did not indicate evidence of significant publication bias (*p* = 0.0916). However, because only 11 studies were included, the ability of funnel-plot-based and regression-based methods to detect publication bias or small-study effects was limited.

### 3.7 Certainty of evidence

The certainty of evidence for the outcome “fermented dairy consumption and the risk of childhood asthma/wheeze” was assessed using the GRADE system. Because all included studies were observational, the initial certainty was rated as low. Based on GRADE criteria, the evidence was downgraded for the following reasons: (1) Inconsistency: substantial heterogeneity was observed across studies (*I*^2^ = 77%, *p* < 0.001), meeting the criteria for serious inconsistency; (2) Imprecision: the 95% confidence intervals in some subgroup analyses crossed the null value (OR = 1), the optimal information size was not achieved, and the effect estimates were characterized by considerable uncertainty; (3) study limitations/robustness concerns: sensitivity analyses showed that the pooled effect was no longer significant after excluding certain studies, suggesting limited stability of the findings. Overall, the certainty of evidence was rated as very low according to the GRADE framework.

## 4 Discussion

### 4.1 Main findings and sources of heterogeneity

This systematic review and meta-analysis synthesized evidence from multiple observational studies to investigate the association between fermented dairy consumption and the risk of childhood asthma and wheeze. The overall pooled analysis showed a statistically significant association between fermented dairy consumption and a reduced risk of the combined outcome of childhood asthma/wheeze (OR = 0.72, 95% CI: 0.55–0.95), but with substantial heterogeneity (*I*^2^ = 77.0%). This heterogeneity is likely attributable to methodological differences across studies, including variations in study design, population characteristics, exposure assessment methods, and the covariates controlled for in individual studies (45). The leave-one-out analysis further indicated that the statistical significance of the primary pooled estimate was sensitive to the exclusion of several individual studies. These studies differed in study design, sample size, exposure classification, outcome assessment, and confounder adjustment. For example, Levin 2019 reported univariate estimates only, and Fsadni 2018 was a small cross-sectional study with limited confounder adjustment, and Jamalvandi 2024 was a large cross-sectional study assessing fermented milk products, whereas Miyake 2009 and Miyake 2014 were cohort studies with relatively more comprehensive covariate adjustment. This sensitivity further supports a cautious interpretation of the overall pooled association. More broadly, exposure assessment was primarily based on food frequency questionnaires, which may introduce recall and measurement bias. Future studies should employ more precise and standardized dietary assessment methods to improve comparability and evidence quality.

### 4.2 Interpretation of subgroup analyses and potential mechanisms

#### 4.2.1 Subgroup analysis by study design

Given the substantial heterogeneity across studies, we performed subgroup analyses based on study design, which revealed marked methodological differences in the observed associations. In cross-sectional studies, fermented dairy consumption was statistically significantly associated with a reduced risk of childhood asthma/wheeze-related outcomes (pooled OR = 0.51, 95% CI: 0.33–0.78), which is consistent with the inverse association suggested by some previous studies (27). In contrast, this association was not observed in prospective cohort studies, which have a clearer temporal relationship between exposure and outcome (46). This discrepancy suggests that the observed inverse association may be partly driven by methodological differences between study designs rather than reflecting a robust causal relationship. One plausible explanation is reverse causation inherent to cross-sectional studies (47), whereby parents of children with wheeze may be more likely to increase their child’s consumption of fermented dairy or probiotic-containing foods. In addition, a probiotic-related health halo effect–whereby fermented dairy products are perceived as intrinsically health-promoting—may have contributed to the stronger inverse association observed in cross-sectional studies. Caregivers who perceive fermented dairy or probiotic-containing products as beneficial may be more likely to report their use and may also engage in other health-conscious behaviors, which could confound the observed association, although these behavioral pathways were not directly assessed in the included studies (48, 49) Residual confounding and exposure misclassification may also contribute to the observed inconsistencies. The significant divergence in effect estimates across study designs highlights the importance of well-designed prospective cohort studies in future research. Overall, because the inverse association was observed primarily in cross-sectional studies, which are more susceptible to reverse causation and residual confounding, the primary pooled estimate should be interpreted cautiously and should not be regarded as evidence of a causal protective effect (50). The exploratory geographic subgroup analysis further suggested possible regional variation in the observed associations. However, this finding should not be interpreted as evidence of an independent geographic effect. The apparent geographic difference may partly reflect the study-design heterogeneity described above, because European studies were predominantly prospective cohort studies, whereas non-European studies included a higher proportion of cross-sectional studies. In addition, the non-European category combined geographically and culturally diverse settings, including Asia, Oceania, and Africa. Therefore, geographic subgroup findings should be interpreted as exploratory and hypothesis-generating, and may reflect differences in study design, background dietary patterns, types of fermented dairy products consumed, exposure assessment methods, and outcome definitions.

#### 4.2.2 Subgroup analysis by outcome subtype

In the subgroup analysis by outcome subtype, this study observed a statistically significant association between fermented dairy consumption and a reduced risk of wheeze in children (OR = 0.62, 95% CI: 0.39–0.98), whereas no statistically significant association was observed for physician-diagnosed asthma (OR = 0.94, 95% CI: 0.76–1.18). However, the difference between the two outcomes did not reach statistical significance (*p* = 0.105), suggesting that the current evidence is insufficient to support a clear phenotype-specific effect. Nevertheless, this difference has some biological plausibility. Wheeze in early childhood is often clinically heterogeneous and is frequently associated with viral infections, airway immaturity, or transient immune dysregulation (51, 52). In contrast, asthma is a chronic inflammatory airway disease whose development involves more complex genetic and environmental factors (53). Therefore, fermented dairy consumption may be more likely to influence short-term or reversible respiratory symptoms such as wheeze, while having a limited impact on the long-term chronic condition of asthma. Moreover, misclassification between wheeze and asthma is common in epidemiological studies (54), and many studies do not strictly distinguish between the two, which may dilute or obscure true associations in statistical analyses. It should be noted that, as the difference between subgroups did not reach statistical significance, the above interpretation remains exploratory and requires further validation in large-scale prospective studies with standardized outcome definitions. Because the composite outcome combined clinically distinct respiratory outcomes, the outcome-specific estimates (Figure 3) provide clinically relevant context and should be interpreted alongside the primary pooled estimate.

#### 4.2.3 Subgroup analysis by exposure window

In the subgroup analysis by timing of exposure, the developmental window of exposure may be important for interpreting these findings. The development of allergic diseases may differ depending on whether exposure occurs prenatally or postnatally, as both periods can influence immune system development through distinct biological mechanisms (55–58). The studies included in this meta-analysis assessed both prenatal exposure (maternal intake during pregnancy) and postnatal exposure (direct intake during childhood). Although our subgroup analysis did not detect a statistically significant difference between the two exposure windows (*p* = 0.598), prenatal exposure was not significantly associated with the outcomes, whereas postnatal childhood intake was associated with a reduced risk. Differences in the timing and duration of exposure across studies may contribute to the observed heterogeneity. From a biological perspective, a role of exposure timing is plausible. Early childhood is a critical period for immune system development and gut microbiota establishment, and dietary factors may participate in immune regulation by modulating the gut microbiome and its metabolites (59, 60). In contrast, prenatal dietary exposure acts indirectly on the fetus primarily through the maternal–placental pathway, a more complex route that may be more difficult to detect reliably in epidemiological studies (61, 62). However, given the lack of statistically significant subgroup differences, these interpretations remain exploratory and hypothesis-generating, and require confirmation in large-scale prospective studies with standardized and harmonized exposure assessment methods.

#### 4.2.4 Subgroup analysis by product type

In the subgroup analysis of different fermented dairy products, only cheese consumption showed a statistically significant association, while yogurt and fermented milk products did not yield statistically significant associations. However, the difference among product types did not reach statistical significance (*p* = 0.484), suggesting that the current evidence is insufficient to support a clear product-specific effect. Nevertheless, different fermented dairy products may not be biologically interchangeable. Yogurt is typically characterized by live lactic acid bacteria and a relatively short fermentation process, whereas aged cheeses undergo longer ripening, during which proteolysis and microbial metabolism can generate a broader range of bioactive peptides, free amino acids, organic acids, and other microbial metabolites (63, 64). These product-specific differences may influence the viability of microorganisms, the structure of the dairy matrix, the release of bioactive peptides, and the availability of fermentation-derived metabolites, all of which could plausibly affect gut microbial activity, epithelial barrier function, and immune-inflammatory signaling (65, 66). Moreover, the exposure category of “fermented dairy products” is often broadly defined in epidemiological studies, with differences in definition, classification, and assessment methods across studies. Such imprecision in exposure classification may dilute potentially subtle effects in statistical analyses (12, 67, 68). Accordingly, the observed differences across product types may reflect both methodological heterogeneity and genuine product-level biological variation; however, because the test for subgroup differences was not statistically significant and the included studies did not directly assess microbiota composition, specific microbial strains, or fermentation-derived metabolites, these product-specific mechanistic interpretations remain exploratory. The primary pooled estimate should therefore be interpreted as a summary association for fermented dairy products as a broad dietary exposure. Future studies should adopt more refined and standardized classifications of dairy products and consider strain-specific and processing-specific analyses.

### 4.3 Limitations and future directions

First, all included studies were observational in design. According to the GRADE evidence grading criteria, the certainty of the evidence was rated as very low, and causal relationships cannot be established. Considerable heterogeneity across studies in terms of populations, exposure assessment methods, and outcome definitions, together with the pooling of different fermented dairy products and the use of a composite asthma/wheeze outcome, may limit the generalizability and clinical specificity of the findings. In addition, some subgroup analyses included multiple stratum-specific estimates from the same study, and because within-study correlations were unavailable, this potential non-independence may have affected the precision of subgroup-specific pooled estimates.

Second, variability in exposure assessment methods and incomplete adjustment for confounding factors may have introduced bias. Furthermore, potential unmeasured confounders cannot be excluded as influencing the results. Sensitivity analyses restricted to higher-quality studies or excluding minimally adjusted studies attenuated the pooled estimate and rendered the association statistically non-significant. In addition, although Egger’s test did not indicate statistically significant publication bias, the small number of included studies limited the ability to detect publication bias or small-study effects; moreover, the statistically significant inverse association observed with the DerSimonian–Laird model was not retained after Hartung–Knapp adjustment. Therefore, the pooled estimates should be interpreted as suggestive rather than statistically robust evidence.

Third, although microbiota modulation, specific microbial strains, and fermentation-derived metabolites were discussed as biologically plausible mechanisms, the included studies did not directly assess microbiota composition, strain-specific effects, or metabolomic profiles; therefore, these explanations should be regarded as hypothesis-generating.

Finally, future research should prioritize well-characterized high-risk pediatric populations, employ robust prospective cohort designs, and incorporate detailed assessments of fermented dairy products, microbial composition, fermentation-derived metabolites, intake levels, and processing characteristics. Such targeted investigations will help clarify the potential role of fermented dairy products as modifiable dietary factors and support the development of evidence-based nutritional and public health strategies.

## 5 Conclusion

In conclusion, this study did not observe a consistent association between fermented dairy consumption and the risk of childhood asthma or wheeze. Although some subgroup analyses suggested stratum-specific associations under specific conditions, the relevant results were not statistically robust—for example, the statistical significance of the primary inverse association was not retained after Hartung–Knapp adjustment—and should be interpreted with caution. In particular, the inconsistency between cohort and cross-sectional studies suggests that the observed potential association may be influenced by methodological factors such as study design. Given the observational nature of the included studies and the overall very low certainty of the evidence, causal relationships cannot be inferred at present. High-quality, large-scale prospective studies are still needed to further clarify this association.

## Data Availability

The data analyzed in this study are derived from previously published studies, which are cited in the article. Extracted data supporting the findings are available in the Supplementary material. Further inquiries can be directed to the corresponding author.
