# Supplementary material for: Association of fermented dairy consumption with asthma and wheeze risk in children: a systematic review and meta-analysis of observational studies
Source: Front Nutr. 2026 Jul 21;13:1860222. doi: 10.3389/fnut.2026.1860222 (PMC13433506; doi:10.3389/fnut.2026.1860222)
Supplement: Supplementary file 1 [file Table_1.DOCX]

**Supplementary** **material**

**Supplementary Table 1-4: Details of the Literature Search Strategy**

**Supplementary Table 1: PubMed**

| No. | Search query |
| --- | --- |
| #1 | ("Child"[Mesh] OR "Infant"[Mesh] OR "Adolescent"[Mesh] OR Child*[Title/Abstract] OR Children [Title/Abstract] OR Pediatric*[Title/Abstract] OR Paediatric*[Title/Abstract] OR Infant*[Title/Abstract] OR Adolescen*[Title/Abstract] OR Toddler*[Title/Abstract]) |
| #2 | ("Dairy Products"[Mesh] OR "Cultured Milk Products"[Mesh] OR "Milk"[Mesh] OR "Cheese"[Mesh] OR Dairy[Title/Abstract] OR Milk[Title/Abstract] OR Yogurt*[Title/Abstract] OR Yoghurt*[Title/Abstract] OR Cheese*[Title/Abstract] OR Fermented milk[Title/Abstract] OR Cultured milk[Title/Abstract] OR Kefir[Title/Abstract] OR Probiotic milk[Title/Abstract] OR Farm milk[Title/Abstract] OR Raw milk[Title/Abstract]) |
| #3 | ("Asthma"[Mesh] OR "Respiratory Sounds"[Mesh] OR "Respiratory Hypersensitivity"[Mesh] OR Asthma*[Title/Abstract] OR Wheez*[Title/Abstract] OR Bronchial hyperreactivity [Title/Abstract]) |
| #4 | #1 AND #2 AND #3 |

**Supplementary Table 2: Web of Science**

| No. | Search query |
| --- | --- |
| #1 | TS=("Child" OR "Infant" OR "Adolescent" OR Child* OR Children OR Pediatric* OR Paediatric* OR Infant* OR Adolescen* OR Toddler*) |
| #2 | TS=("Dairy Products" OR "Cultured Milk Products" OR "Milk" OR "Cheese" OR Dairy OR Milk OR Yogurt* OR Yoghurt* OR Cheese* OR "Fermented milk" OR "Cultured milk" OR Kefir OR "Probiotic milk" OR "Farm milk" OR "Raw milk") |
| #3 | TS=("Asthma" OR "Respiratory Sounds" OR "Respiratory Hypersensitivity" OR Asthma* OR Wheez* OR "Bronchial hyperreactivity") |
| #4 | #1 AND #2 AND #3 |

**Supplementary Table 3: Embase**

| No. | Search query |
| --- | --- |
| #1 | ('children' OR 'child'):ti,ab |
| #2 | ('asthma bronchiale' OR 'asthma pulmonale' OR 'asthma, bronchial' OR 'asthmatic' OR 'asthmatic subject' OR 'bronchial asthma' OR 'bronchus asthma' OR 'chronic asthma' OR 'lung allergy' OR 'asthma'):ti,ab |
| #3 | ('sibilant respiration' OR 'sibilant rhonchi' OR 'wheeze' OR 'wheezing'):ti,ab |
| #4 | ('cultured dairy foods' OR 'cultured dairy product' OR 'cultured milk foods' OR 'cultured milk product' OR 'cultured milk products' OR 'fermented dairy foods' OR 'fermented milk' OR 'fermented milk product' OR 'fermented dairy product'):ti,ab |
| #5 | ('yoghourt' OR 'yogurt' OR 'zabadi' OR 'yoghurt'):ti,ab |
| #6 | ('cheddar' OR 'cheddar cheese' OR 'cheese'):ti,ab |
| #7 | ('kephir' OR 'kefir'):ti,ab |
| #8 | #4 OR #5 OR #6 OR #7 |
| #9 | #1 AND (#2 OR #3) AND #8 |

**Supplementary Table 4: Cochrane**

| No. | Search query |
| --- | --- |
| #1 | MeSH descriptor: [Child] explode all trees |
| #2 | (children):ti,ab,kw OR (child):ti,ab,kw |
| #3 | #1 OR #2 |
| #4 | MeSH descriptor: [Asthma] explode all trees |
| #5 | (Asthmas):ti,ab,kw OR (asthma):ti,ab,kw |
| #6 | MeSH descriptor: [Respiratory Sounds] explode all trees |
| #7 | (Rub, Pleural):ti,ab,kw OR (Pleural Rub):ti,ab,kw OR (Pleural Rubs):ti,ab,kw OR (Respiratory Sound):ti,ab,kw OR (Sounds, Breathing):ti,ab,kw |
| #8 | (Sound, Breathing):ti,ab,kw OR (Sound, Lung):ti,ab,kw OR (Sounds, Lung):ti,ab,kw OR (Lung Sound):ti,ab,kw OR (Sound, Respiratory):ti,ab,kw |
| #9 | (Breathing Sound):ti,ab,kw OR (Breathing Sounds):ti,ab,kw OR (Lung Sounds):ti,ab,kw OR (Wheezings):ti,ab,kw OR (Wheezing):ti,ab,kw |
| #10 | (Stridor):ti,ab,kw OR (Stridors):ti,ab,kw OR (Crackle):ti,ab,kw OR (Rale):ti,ab,kw OR (Rales):ti,ab,kw |
| #11 | (Crackles):ti,ab,kw OR (Rhonchi):ti,ab,kw OR (Rhonchus):ti,ab,kw OR (Respiratory Sounds):ti,ab,kw |
| #12 | #4 OR #5 OR #6 OR #7 OR #8 OR #9 OR #10 OR #11 |
| #13 | MeSH descriptor: [Cultured Milk Products] explode all trees |
| #14 | (Cultured Milk Products):ti,ab,kw OR (Products, Fermented Milk):ti,ab,kw OR (Dairy Products, Fermented):ti,ab,kw OR (Fermented Dairy Product):ti,ab,kw OR (Milk Products, Cultured):ti,ab,kw |
| #15 | (Product, Fermented Milk):ti,ab,kw OR (Fermented Milk Products):ti,ab,kw OR (Milk Products, Fermented):ti,ab,kw OR (Products, Fermented Dairy):ti,ab,kw OR (Dairy Product, Fermented):ti,ab,kw |
| #16 | (Fermented Dairy Products):ti,ab,kw OR (Product, Cultured Milk):ti,ab,kw OR (Product, Fermented Dairy):ti,ab,kw OR (Milk Product, Fermented):ti,ab,kw OR (Fermented Milk Product):ti,ab,kw |
| #17 | (Milk Product, Cultured):ti,ab,kw OR (Cultured Milk Product):ti,ab,kw OR (Products, Cultured Milk):ti,ab,kw |
| #18 | #13 OR #14 OR #15 OR #16 OR #17 |
| #19 | MeSH descriptor: [Yogurt] explode all trees |
| #20 | (Yoghurt):ti,ab,kw OR (Yogurt):ti,ab,kw |
| #21 | MeSH descriptor: [Kefir] explode all trees |
| #22 | (Kefir Grains):ti,ab,kw OR (Grains, Kefir):ti,ab,kw OR (Grain, Kefir):ti,ab,kw OR (Kefir Grain):ti,ab,kw OR (Kefir):ti,ab,kw |
| #23 | #18 OR #19 OR #20 OR #21 OR #22 |
| #24 | #3 AND #12 AND #23 |

**Supplementary Table 5. Quality assessment of cohort and case–control studies using the Newcastle–Ottawa Scale (NOS)**

|  | Miyake 2009 | Maslova  2012 | Miyake  2014 | Bertelsen 2014 | Stampfli 2022 | Koivusaari 2022 | Nicklaus 2019 |
| --- | --- | --- | --- | --- | --- | --- | --- |
| **Selection** |  |  |  |  |  |  |  |
| 1.Representativeness of the exposed cohort | * | * | * | * | * |  | * |
| 2. Selection of the non-exposed cohort | * | * | * | * | * | * | * |
| 3. Ascertainment of exposure |  |  |  |  |  |  |  |
| 4. Demonstration that outcome of interest was not present at start of study | * | * | * | * | * | * | * |
| **Comparability** |  |  |  |  |  |  | * |
| 1. Comparability of cohorts on the basis of the design or analysis | ** | ** | ** | ** | ** | ** | ** |
| **Outcome** |  |  |  |  |  |  | * |
| 1. Assessment of outcome |  | * |  |  |  | * | * |
| 2. Was follow-up long enough for outcomes to occur | * | * | * | * | * | * | * |
| 3. Adequacy of follow up of cohorts | * | * | * | * | * | * |  |
| **Score** | 7 | 8 | 7 | 7 | 7 | 7 | 7 |

**Supplementary Table 6. Quality assessment of cross-sectional studies using a modified version of the Newcastle–Ottawa Scale (NOS)**

|  | K. Wickens 2002 | Jamalvandi 2024 | Levin 2019 | Fsadni 2018 |
| --- | --- | --- | --- | --- |
| Selection |  |  |  |  |
| 1. Representativeness of the sample | * | * | * | * |
| 2. Sample size | * | * | * |  |
| 3. Non-respondents |  | * | * |  |
| 4. Ascertainment of the exposure (risk factor) | ** | * | ** | * |
| Comparability |  |  |  |  |
| 1. The subjects in different outcome groups are comparable, based on the study design or analysis. Confounding factors are controlled. | ** | ** | * | ** |
| Outcome |  |  |  |  |
| 1. Assessment of outcome | * | * | * | * |
| 2. Statistical test | * | * | * | * |
| **Score** | 8 | 9 | 8 | 6 |

**Supplementary Table 7. Comparison of DerSimonian–Laird and Hartung–Knapp results for the primary and study–design–stratified analyses.**

| **Analysis** | **k** | **DerSimonian–Laird OR** | **DerSimonian–Laird  95% CI** | **DerSimonian–Laird  *P* value** | **Hartung–Knapp  OR** | **Hartung–Knapp  95% CI** | **Hartung–Knapp  P value** | **q** | **df** |
| --- | --- | --- | --- | --- | --- | --- | --- | --- | --- |
| Overall | 11 | 0.72 | 0.55–0.95 | 0.018 | 0.72 | 0.52–1.01 | 0.054 | 1.18 | 10 |
| Cohort studies | 7 | 0.91 | 0.72–1.16 | 0.462 | 0.91 | 0.64–1.30 | 0.555 | 1.39 | 6 |
| Cross-sectional studies | 4 | 0.51 | 0.33–0.78 | 0.002 | 0.51 | 0.23–1.13 | 0.073 | 1.31 | 3 |

Note: The Hartung–Knapp adjustment was applied to the DerSimonian–Laird estimate of between-study variance. Hartung–Knapp P values were computed using a t distribution with k-1 degrees of freedom. q, scaling factor; df, degrees of freedom.

**Supplementary Table 8. Sensitivity analyses excluding studies with lower methodological quality or minimal confounder adjustment.**

| **Analysis** | **Excluded studies** | **k** | **OR** | **95%CI** | ***P* for pooled effect** | **I²** |
| --- | --- | --- | --- | --- | --- | --- |
| Primary analysis | None | 11 | 0.72 | 0.55–0.95 | 0.018 | 77% |
| Restricting to studies with NOS ≥7 | Fsadni et al. (2018) | 10 | 0.78 | 0.61–1.01 | 0.058 | 72.6% |
| Excluding minimally adjusted studies | Levin et al. (2019);  Fsadni et al. (2018) | 9 | 0.85 | 0.67–1.07 | 0.172 | 66.4% |

Note: Analyses in this table used the DerSimonian–Laird random-effects model for comparability with the primary analysis.

**Supplementary Figure 1.** **Leave‑one‑out sensitivity analysis.**

**Supplementary Figure 2. Forest plot after excluding RR/HR studies**

**Supplementary Figure 3. Trim‑and‑fill funnel plot.**

**Supplementary Figure 4.** **Studies with minimal confounder adjustment were excluded, including Levin et al. (2019) and Fsadni et al. (2018). The pooled estimate was calculated using the DerSimonian–Laird random-effects model and stratified by study design.**

**Supplementary Figure 5. Exploratory subgroup analysis by geographic region. Studies were grouped as European and non-European studies because several individual regions were represented by only one or a few studies. The pooled estimates were calculated using the DerSimonian–Laird random-effects model.**
